# Highly efficient and precise base editing by engineered dCas9-guide tRNA adenosine deaminase in rats

**DOI:** 10.1038/s41421-018-0047-9

**Published:** 2018-07-17

**Authors:** Yuanwu Ma, Lei Yu, Xu Zhang, Changpeng Xin, Shisheng Huang, Lin Bai, Wei Chen, Ran Gao, Jing Li, Shuo Pan, Xiaolong Qi, Xingxu huang, Lianfeng Zhang

**Affiliations:** 1 Key Laboratory of Human Disease Comparative Medicine, NHFPC, Institute of Laboratory Animal Science, Peking Union Medicine College, Chinese Academy of Medical Sciences, Beijing, 100021 China; 2 Neuroscience Center, Chinese Academy of Medical Sciences, Beijing, 100021 China; 3 Bio-Med Big Data Center Omics Core Facility, CAS-MPG Partner Institute of Computational Biology, Shanghai, 200031 China; 4 School of Life Science and Technology, Shanghai Tech University, Shanghai, 201210 China

Dear Editor

Rats are reference laboratory animal models for understanding mechanism of human diseases such as diabetes, hypertension, and neurological disorder^1,2^. CRISPR/Cas9 system has proved an efficient and flexible tool to generate gene modified rats^3–6^. Great efforts have been made to reduce the side effects and extend the application of this system^7–10^. Cytosine base editor (CBE), containing the engineered cytosine deaminase with CRISPR/Cas9 can be used to modify mammal genomic DNA without induction of double-strand DNA break or template. CBE targets sequence by inducing C·G to T·A conversion with a window of approximately five nucleotides^11^. Recently, the same group developed a new dCas9-guide tRNA adenosine deaminase which was capable of inducing A·T to G·C conversion. Together with CBE, ABE enables introduction of all four nucleotides transitions (C to T, A to G, T to C, and G to A) in target genomic sequence^12^. These base-editing tools provide a much safer approach compared with wild-type CRISPR/Cas9 system for gene correction of human disease. Here we report the application of this newly developed adenine base editors in rat base editing.

Two experiments were designed to test this system in rats. For the first experiment, we selected one targeting site at *Hemgn* gene locus (Fig. 1, Supplementary Table S1). For the second experiment, we selected two targeting sites at *N-deacetylase* and *N-sulfotransferase 4* (*Ndst4*) gene loci (Fig. 1, Supplementary Table S1). The ABE and sgRNAs were prepared in vitro as described^4,12^. Twenty-five nanograms per microliter of ABE mRNA and 20 ng/ml of sgRNA were prepared for microinjection^4^. Animals in Sprague Dawley (SD) background were used in all experiments. Tail genomic DNA of born pups was extracted and used as a template for genotyping.

**Fig. 1:**
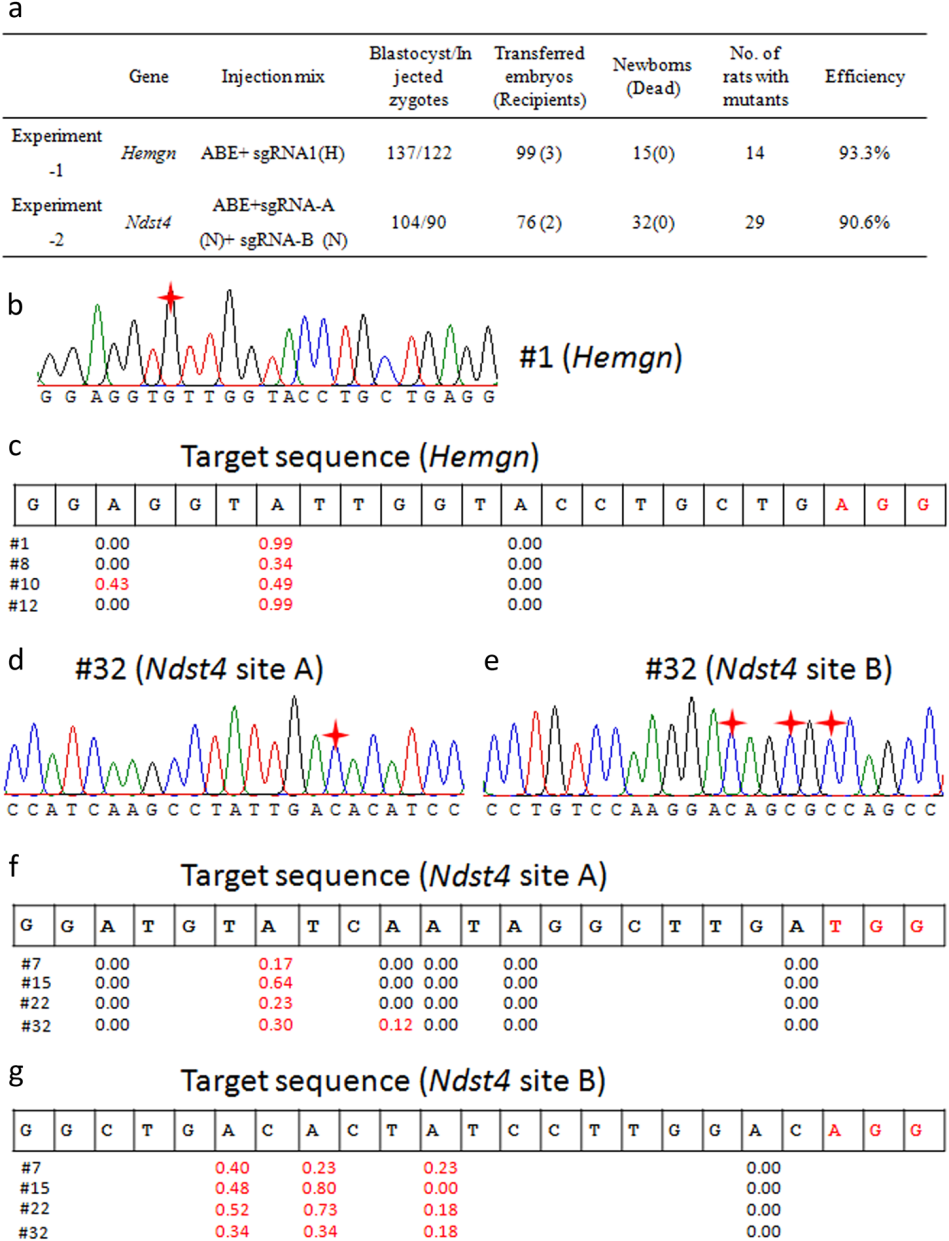
Highly efficient base editing using ABE system in rats. **a** Summary of the base editing experiments in rats. **b** The representative sequence chromatogram of *Hemgn* targeting sequence (potential founder #1). The conversion of A to G at target site was marked with red star. **c** Summary of the targeted deep sequencing of on-target site for *Hemgn* (potential founders #1, #8, #10, and #12). The conversion efficiency of every A was indicated. The PAM was highlight in red. **d** The representative sequence chromatogram of *Ndst4*-A targeting sequence (clone #4 of potential founder #32). The conversion of T to C at target site was marked with red star. **e** The representative sequence chromatogram of *Ndst4*-B targeting sequence (clone #4 of potential founder #32). The conversion of T to C at target site was marked with red star. **f** Summary of the targeted deep sequencing of on-target site for *Ndst4-*A (potential founders #7, #15, #22, and #32). The conversion efficiency of every A was indicated. The PAM was highlight in red. **g** Summary of the targeted deep sequencing of on-target site for *Ndst4-*A (potential founders #7, #15, #22, and #32). The conversion efficiency of every A was indicated. The PAM was highlight in red

For *Hemgn* targeting, a total of 99 injected zygotes were transferred to 3 pseudopregnant female SD rats and 15 pups were born (Fig. 1a). To test the base editing efficiency, the fragment including the target site was amplified and sequenced (Supplementary Table S2). Fourteen rats (14/15) (potential founders #1–2, #4–15) contained an A to G conversion at the 14th base distal from the PAM (Fig. 1a, b; Supplementary Fig. S1). Two rats (2/15) (potential founders #5, #10) revealed an A to G conversion at the 18th base distal from the PAM (Fig. 1a, b; Supplementary Fig. S1), indicating the high base-editing efficiency.

To further analyze the on-target editing effects, deep sequencing was applied to sample #1, #8, #10, and #12. More than 3 M (1024×1024 bit) clean data for each sample were obtained (Supplementary Table S4). The result showed that 14th base distal from the PAM showed A to G conversion with efficiency as high as 0.99 for #1, 0.34 for #8, 0.49 for #10, and 0.99 for sample #12. The conversion efficiency at 18th base distal from the PAM was 0.43 for #10 (Fig. 1c). No other alteration or indel was detected in the selected samples (data not shown). The results showed ABE is a highly precise and efficient base editor for rat genome editing.

For most of human diseases with more than one mutation. Multiple base editing capability is valuable for gene therapy. To test this possibility, two sgRNAs targeting different exons at *Ndst4* gene locus were designed in the second experiment (Supplementary Table S1). Ten nanograms of each sgRNA was used for microinjection to avoid toxic effects. The target sites amplified by the same strategy were analyzed and sequenced. For *Ndst4* targeting, a total of 76 injected zygotes were transferred to 2 pseudopregnant female SD rats and 32 pups were born (Fig. 1a). Fifteen rats (15/32) (potential founders #7, #11, #13–15, #17, #19, #21–25, #29–30, and #32) showed an A to G conversion at the 14th base distal from the PAM at targeting site A (Supplementary Fig. S2). Twenty-nine rats (29/32) (potential founders #1–8, #10–25, #27–30, and #32) showed an A to G conversion at targeting site B (Fig. 1a, d, e, Supplementary Fig. S3). In targeting site A, all mutations were occurred at 14th nucleotide distal from PAM. In targeting site B, 5 rats (potential founders #7, #18, #20, #22, and #32) showed triple A to G conversions at the 15th, 13th, 10th nucleotide distal from PAM, 9 rats (potential founders #1, #2, #5, #6, #14, #15, #21, #24, and #27) showed dual A to G conversions at the 15th, 13th nucleotide distal from PAM, 1 rat (potential founder #3) showed dual A to G conversions at the 15th, 10th nucleotide distal from PAM, 13 rats (potential founders #4, #8, #10, #11–13, #16, #17, #19, #23, #25, #28, and #30) showed single A to G conversion at the 15th nucleotide distal from PAM, and 1 rat (potential founder #29) showed single A to G conversion at the 13th nucleotide distal from PAM. Fifteen rats (15/32) (potential founders #7, #11, #13–15, #17, #19, #21–25, #29–30, #32) revealed an A to G conversion at both targeting sites (A and B) simultaneously **(**Fig. 1a, e; Supplementary Figs. S2 & S3, Table S3).

For further analysis, four samples (potential founders #7, #15, #22, and #32) were selected for on-target (site A and site B) analysis by deep sequencing (Supplementary Table S4). The results showed that conversion rate at site A at the 14th nucleotide was about 0.17 for #7, 0.64 for #15, 0.23 for #22, and 0.30 for #32, which was consistent with the results of PCR products sequencing (Fig. 1f). The conversion rate at site B at the 15th nucleotide was about 0.40 for #7, 0.48 for #15, 0.52 for #22, and 0.34 for #32. The conversion rate at site B at the 13th nucleotide was about 0.23 for #7, 0.80 for #15, 0.73 for #22, and 0.34 for #32. The conversion rate at site B at the 10th nucleotide was about 0.23 for #7, 0.00 for #15, 0.18 for #22, and 0.18 for #32 (Fig. 1g).

Genome-editing functions beyond one-cell stage may induce mosaicism. Therefore, we detected whether ABE system causes mosaicism, and all mutant rats were genotyped by TA clone and subsequent sequencing. Our results showed that more than two mutations were detected for *Ndst4-B* (potential founders #1–3, #5, #17, #18, #20, #22, and #32) (Supplementary Fig. S4). No evidence of mosaicism was found for *Hemgn* and *Ndst4-A* (Supplementary Fig. S4). These results indicate that ABE system does induce genetic mosaicism in rats.

In addition, we also tested the transmission of substitutions by crossing 3 F_0_ mutants (potential founders #11 and #13 for *Hemgn*; potential founder #21 for *Ndst4*-A and -B) with wild-type SD rats and determined the genotypes of the F_1_ rats. The PCR products of F_1_ rats were further analyzed by sequencing. Sequencing results showed that the same substitutions as their parent rats appeared in offsprings (Supplementary Fig. S5), demonstrating that ABE-induced substitutions in rats are transmittable.

Testing the off-target effects is very important for evaluating a new genome-editing tool. Here, we examined 11 potential off-target sites (on-target site not included) for *Hemgn* sgRNA in 4 selected samples (potential founders #1, #8, #10, and #12) and 14 off-target sites (on-target site not included) for *Ndst4* sgRNA-A, 14 off-target sites (on-target site not included) for *Ndst4* sgRNA-B in 4 selected samples (potential founders #7, #15, #22, and #32) identified using Cas-OFFinder^13^ (Supplementary Table S4 & S5). All PCR products were sequenced and analyzed. No off-target mutation was found in those samples. To further analyze the off-target effects, we performed targeted deep sequencing on the selected potential off-target sites. As described above, more than 3 M clean reads for each off-target site were obtained. As expected, no off-target effects were detected. We next performed whole-genome sequencing (WGS) with a sequencing depth of ×30 for samples H-8 (*Hemgn* targeting rat), H-10 (*Hemgn* targeting rat), N-15 (*Ndst4-*A and -B targeting rat), and N-32 (*Ndst4-*A and -B targeting rat) to detect ABE-induced off-target effects. Based on Cas-OFFinder, 33,718 potential off-target sites for *Hemgn* sgRNA, 20,774 potential off-target sites for *Ndst4* sgRNA-A, 22,547 potential off-target sites for *Ndst4* sgRNA-B were obtained with up to 2-bp mismatch in seed region and 8-bp mismatch in non-seed region with NRG PAM (Supplementary Fig. S6). Among these potential off-target sites, only several potential off-target sites were obtained from WGS data (Supplementary Fig. S6, Table S6). We further sequenced these potential off-target sites, and no real off-target sites were obtained (data not shown). Taken together, these results showed that ABE system is a reliable genome-editing tool for rat base editing.

We also analyzed the possible indels mediated by ABE at on-target sites and off-target sites. Only very low signals were detected at these sites, this may be caused by noise of deep sequencing (data not shown). Taken together, our results showed that the ABE system induces base conversion in a much safer way compared with traditional CRISPR/Cas9 system.

In summary, our data showed the successful application of ABE in rat base editing. This exciting strategy showed a highly efficient, precise, and safe way to edit the rat genome. Our data demonstrate the potential of ABE in correction of human genetic disease-associated mutations.

## Electronic supplementary material

Supplementary Information
