# Supplementary material for: Highly efficient and precise base editing by engineered dCas9-guide tRNA adenosine deaminase in rats
Source: Cell Discov. 2018 Jul 17;4:39. doi: 10.1038/s41421-018-0047-9 (PMC6048098; doi:10.1038/s41421-018-0047-9)
Supplement: Supplementary file 1 — Supplementary Information [file 41421_2018_47_MOESM1_ESM.docx]

**Supplementary Information**

This Supplementary Information file contains the following contents: Supplementary Methods, Supplementary Figures (1~6), Supplementary Tables (1~6).

**Supplementary Methods**

**Animals**

All rats used in this study were SD background and bred in an AAALAC-accredited facility with standard food and water libitum. All experiments were approved by the Animal Care and Use Committees of the Institute of Laboratory Animal Science of Peking Union Medical College (ILAS-GC-2015-001).

**DNA constructs**

The ABE7.10 plasmid was reported by David R. Liu [1]. The oligonucleotides for the sgRNA construction were synthesized, annealed, and inserted into pUC57-sgRNA vector (Addgene, 51132) as described previously (Table S1) [2].

***In vitro* transcription of ABE and sgRNAs**

*In vitro* transcription of ABE7.10 and sgRNAs were performed as described [2]. In brief, ABE7.10 expression plasmid was extracted and linearized by digestion with *Age* I (NEB, R0552S). The linearized fragement was purified with phenol-chloroform method and used as the template for *in vitro* transcription (Ambion, AM1345). The sgRNA plasmid was extracted and linearized by digestion with *Dra* I (NEB, R0129S). The linearized sample was purified with phenol-chloroform method and used as the template for *in vitro* transcription (Ambion, AM1354). The ABE mRNA and sgRNAs were purified with the MEGAclear Kit (Ambion, AM1908).

**Microinjection sample preparation**

For microinjection, ABE7.10 mRNA (25 ng/μl) and sgRNAs (10 ng/μl/each) were used. All reagent and solution used for microinjection were RNase-free.

**ABE/sgRNA injection into fertilized rat eggs**

All rats were SD background and purchased from Beijing Vital River Laboratories. The microinjection was performed as before [2]. In brief, fertilized rat eggs were obtained from female rats mated with males after treatment with pregnant mare serum gonadotropin (PMSG, Sigma-Aldrich) and human chorionic gonadotropin (hCG, Sigma-Aldrich). Microinjections were performed follow the procedure as described [3]. After microinjection, the injected fertilized rat eggs were transferred to pseudopregnant SD rats.

**Genomic DNA preparation and genotyping**

Genomic DNA was extracted from the tails of 7-day-old rats with an EasyPure Genomic DNA Kit (Transgene biotech, EE101-01). The fragments including the target sites were amplified and sequenced. The PCR primers used for genotyping are listed in Table S2. All the PCR products of the samples were sequenced directly. To detect the exact mutation, the PCR products were sub-cloned for sequencing analysis.

**Targeted deep sequencing**

Shorter sequences (less than 500 bp) containing the on-target sites or off-target sites were amplified with high-fidelity polymerase. All of the used primers were listed in Table S4. All PCR products were purified with a QIAquick Gel Extraction Kit (QIAGEN, 28704). PCR products were submitted to Shanghai Institutes of Biological Sciences for targeted deep sequencing using illumina Hiseq2500 (PE250). Each site with more than 3 M clean reads.

**Whole genome sequencing**

Whole genome sequencing was performed in rat genomic DNA at a sequencing depth of 30× using illumina Hiseq ×10 (PE150). Six samples including wild type and ABE-treated samples were selected for sequencing. We mapped the sequencing data using Isaac aligner with a rat reference genome (Rnor_6.0). Variants were identified by SNP database were excluded from the identified variants. To check the potential off-target effects, we picked out that A and T converted to the other bases among the remained variants. Then we excluded the the common variant both in wild type and ABE-treated samples. The positive sites were compared with the candidates from Cas-OFFinder with up to 2 bp mismatch in seed region and 8 bp mismatch in non-seed region.

**Germline transmission**

The selected potential founder rats were crossed with wild-type SD rats to test the germline transmission. Germline transmission was considered successful when mutants can be detected and identical to that of the founder parent.

**Data availability**

High-throughput sequencing data have been depos­ited in the NODE (<http://www.biosino.org/node/index>) with accession code OEP000027.

**Supplementary Figures**

**
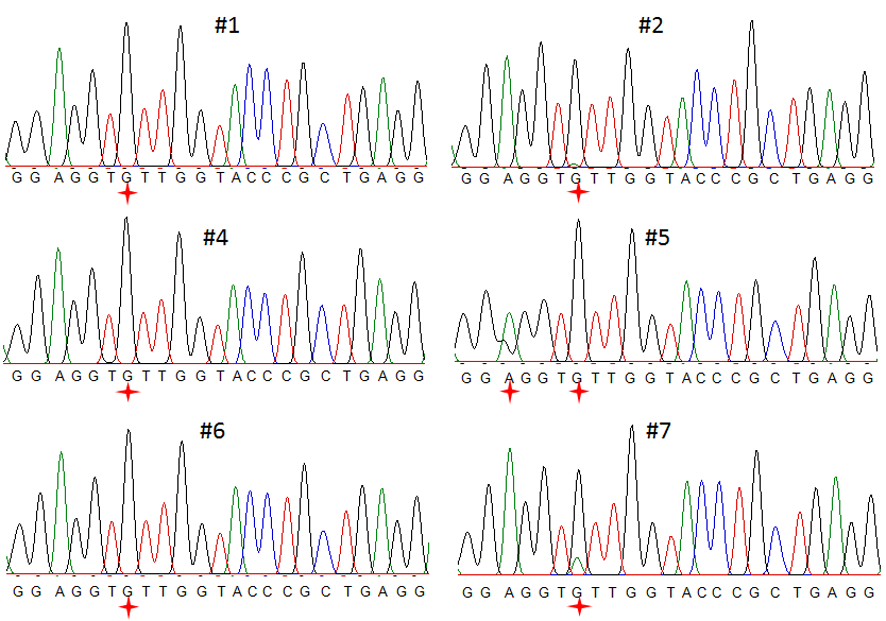
**

**
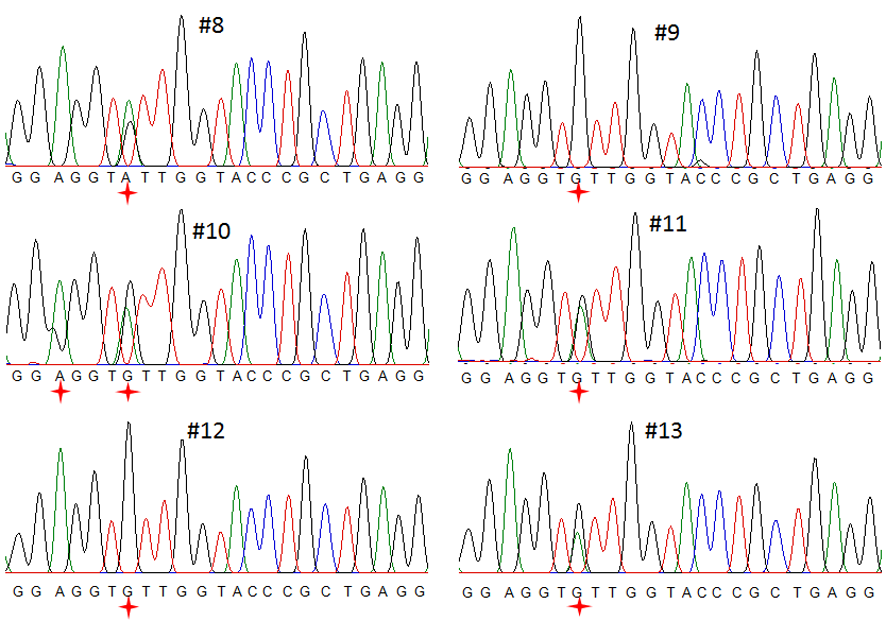
**

**
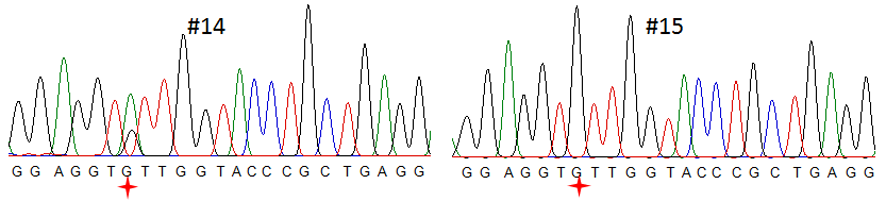
**

**Figure S1 Detection of ABE-mediated base editing in *Hemgn* sgRNA targeting site**

The Chromatogram of *Hemgn* targeting site in PCR samples from Experiment 1. The target sequence was shown. The red stars indicated the conversion of A to G.

**
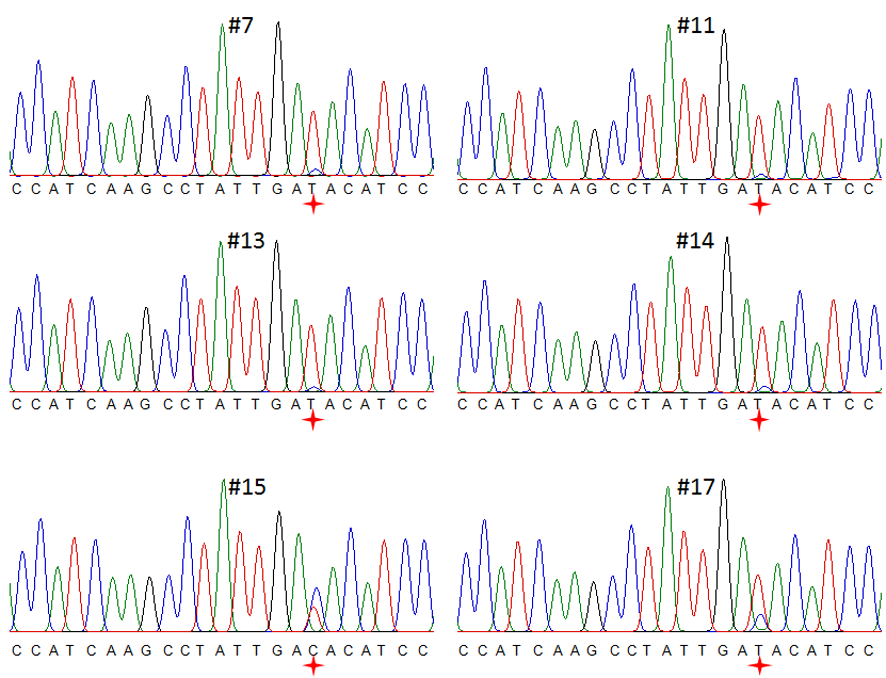
**

**
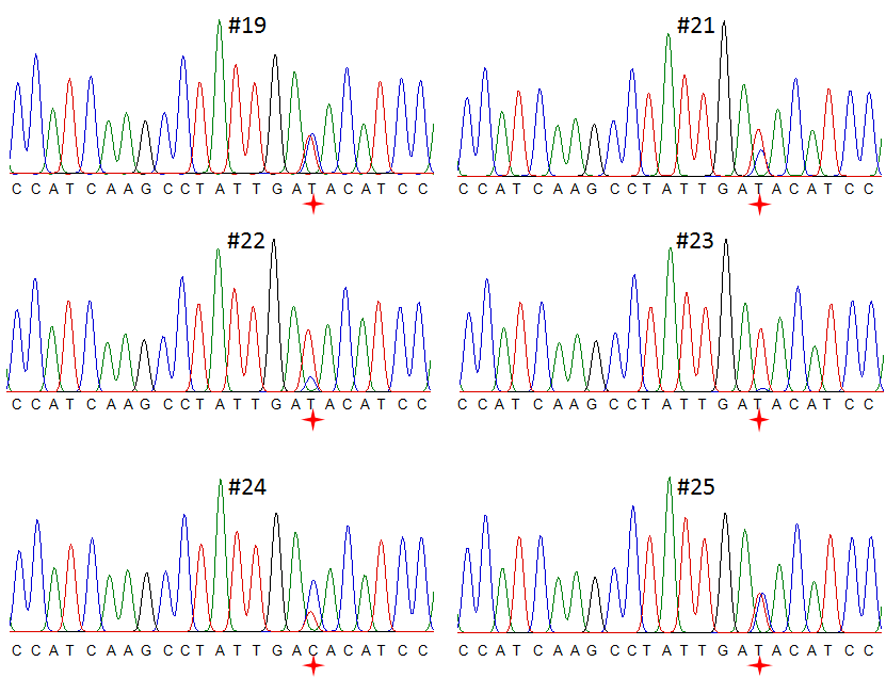
**

**
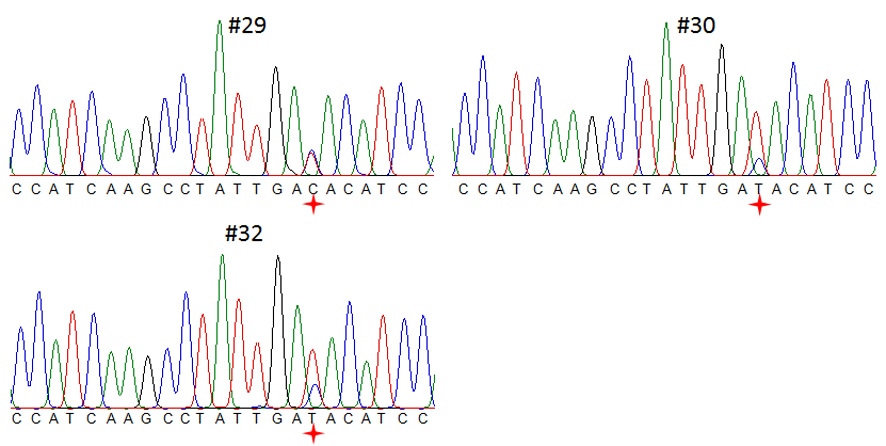
**

**Figure S2 Detection of ABE-mediated base editing in *Ndst4* sgRNA targeting site A**

The Chromatogram of *Ndst4* targeting site A in PCR samples from Experiment 2. The target sequence was shown. The red stars indicated the conversion of T to C.


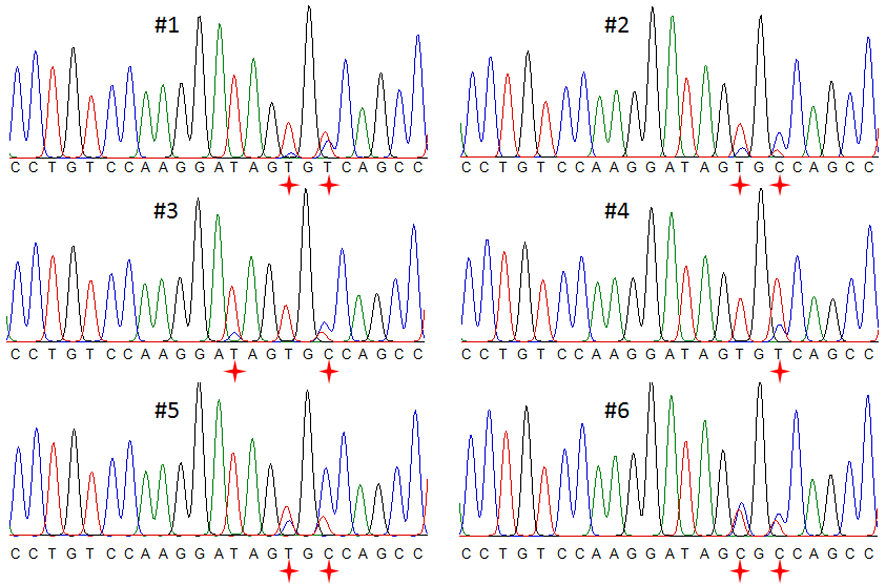


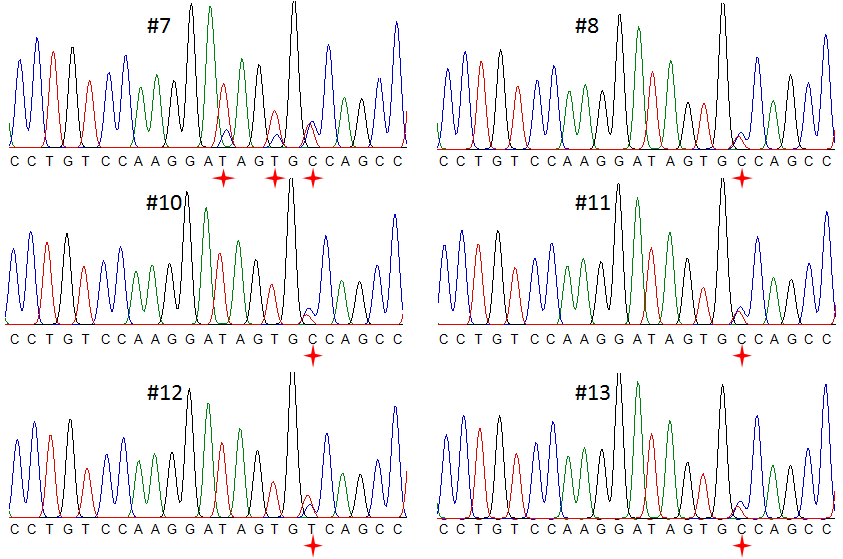


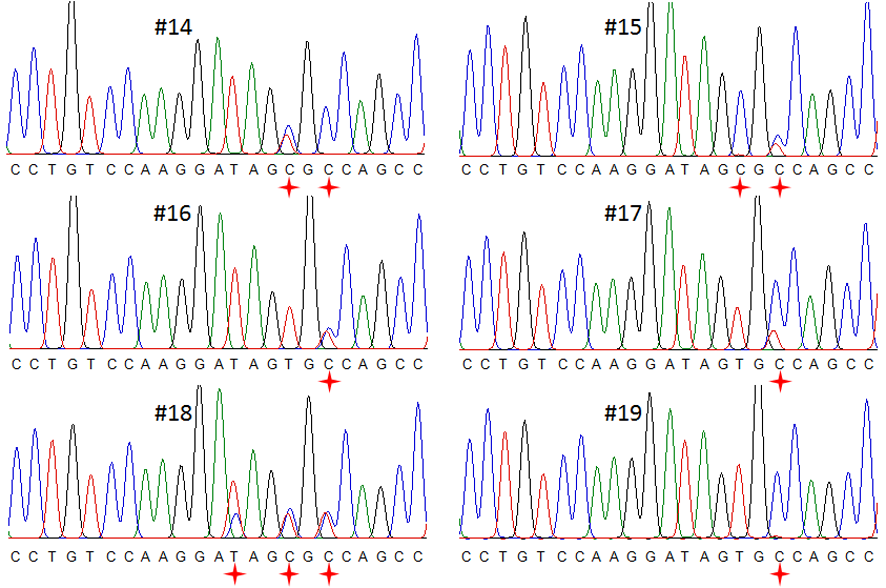


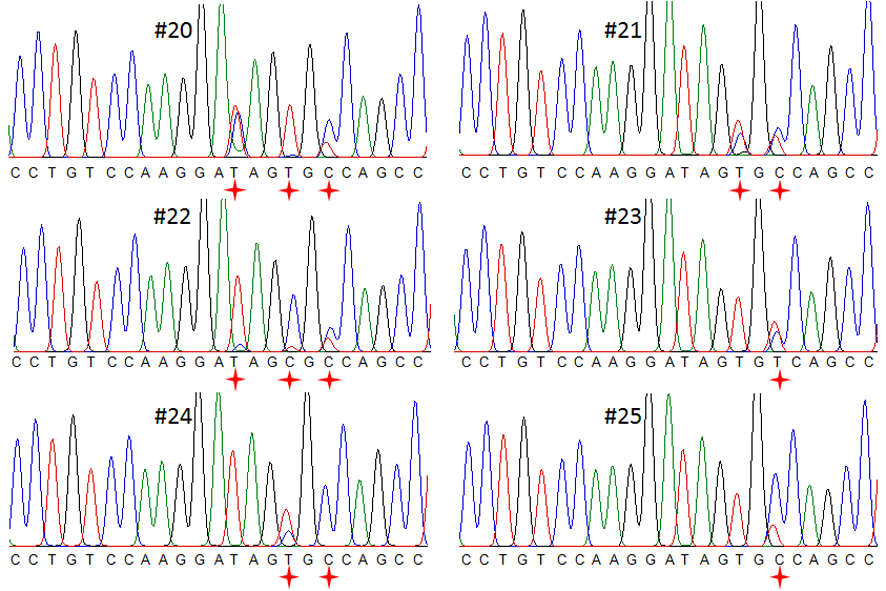


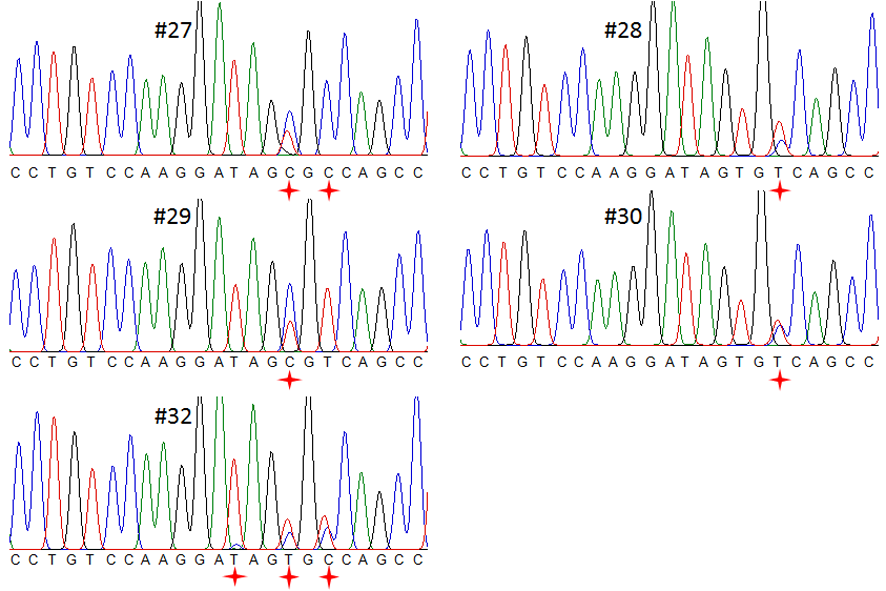


**Figure S3 Detection of ABE-mediated base editing in *Ndst4* sgRNA targeting site B**

The Chromatogram of *Ndst4* targeting site B in PCR samples from Experiment 2. The target sequence was shown. The red stars indicated the conversion of T to C.


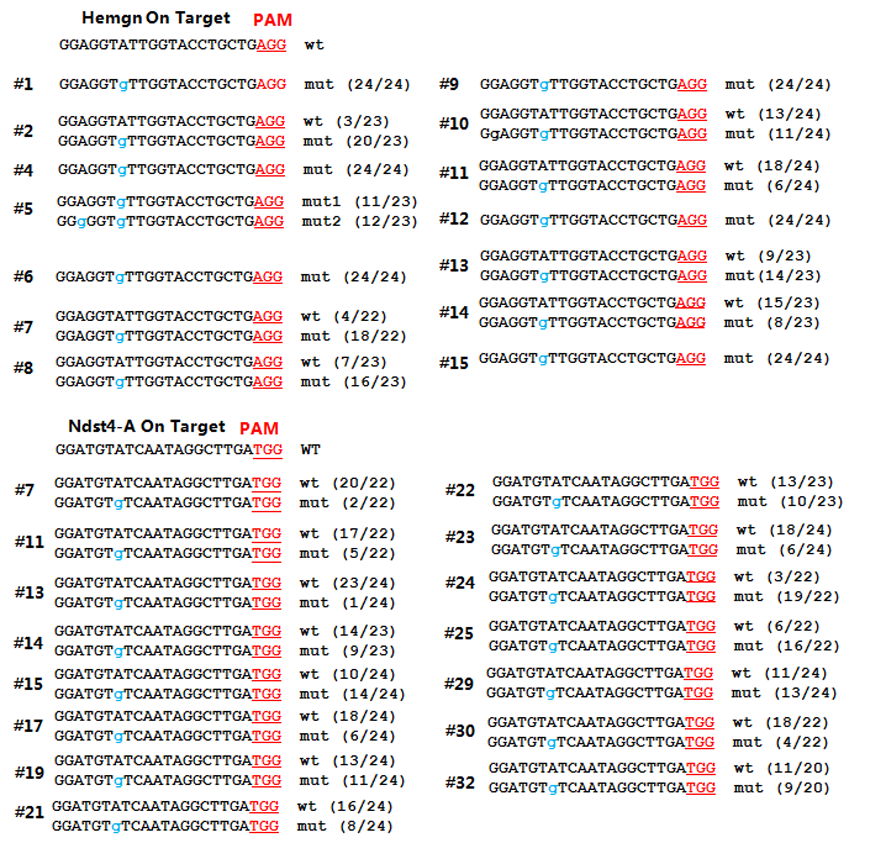


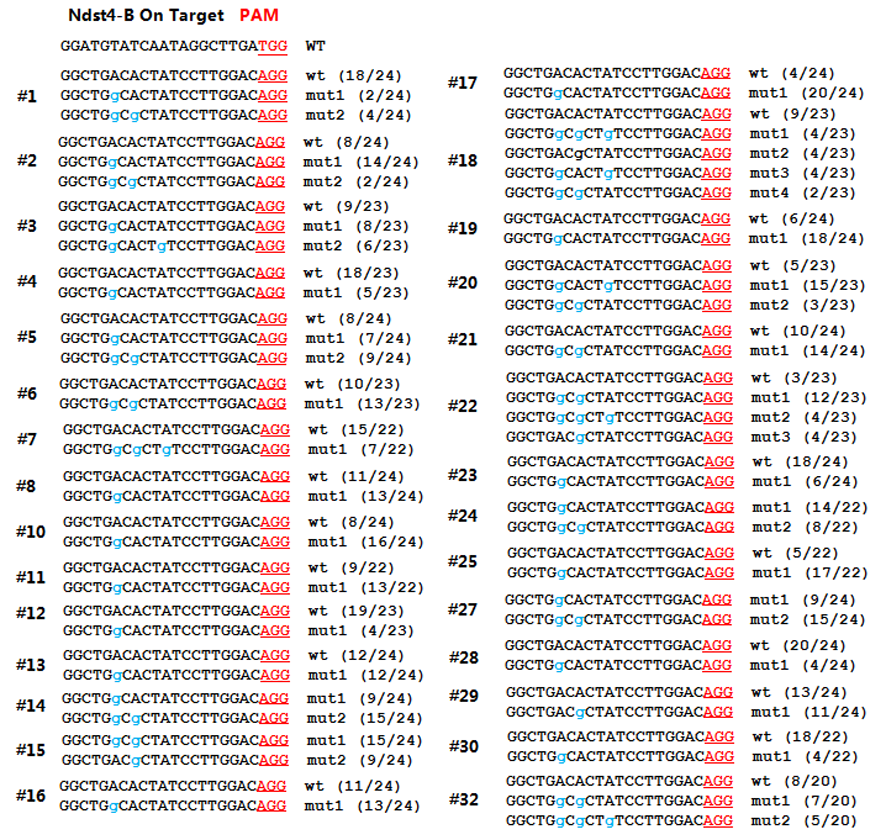


**Figure S4 Sequences of modified *Hemgn* and *Ndst4*-A and -B locus**

All mutant rats were TA cloned and analyzed by Sanger sequencing. The PAM sequence is underlined in red. The substituted nucleotides were shown in blue. N/N represents positive colonies out of total sequenced.


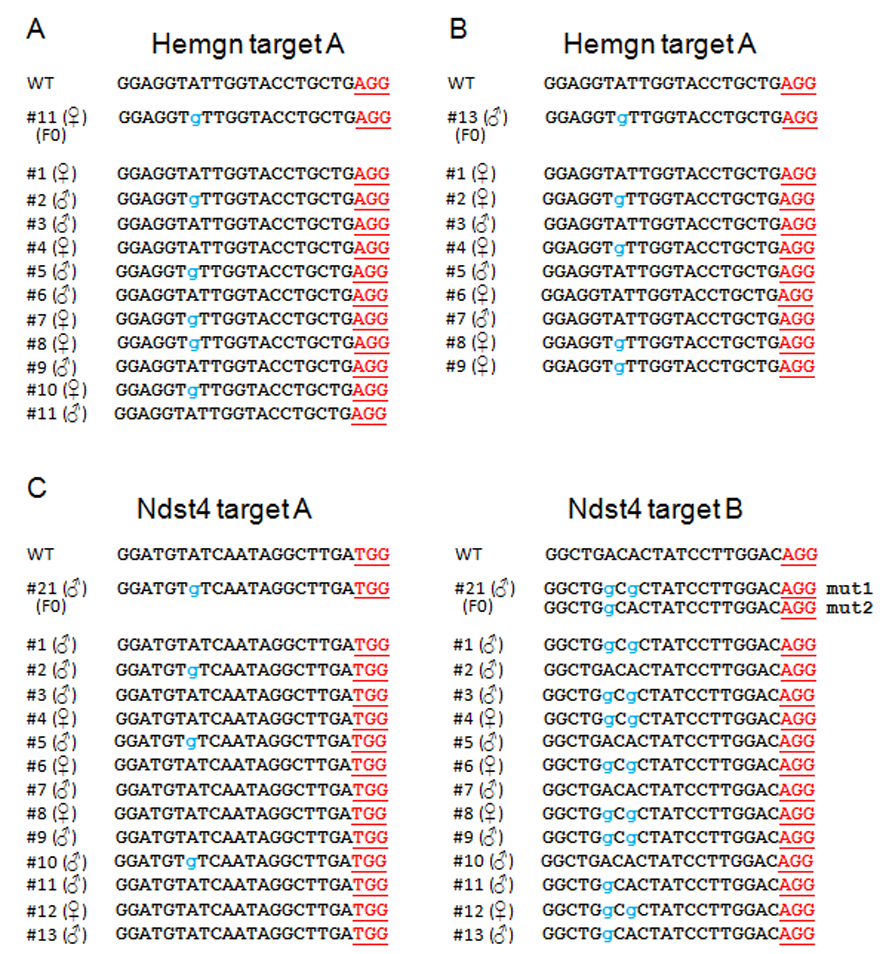


**Figure S5 Germline transmission of the *Hemgn* and *Ndst4* mutant rats.**

(A) Genotyping the F_1_ rats obtained by crossing *Hemgn* mutant rat (founder #11) with wild type rat.

(B) Genotyping the F_1_ rats obtained by crossing *Hemgn* mutant rat (founder #11) with wild type rat.

(C) Genotyping the F_1_ rats obtained by crossing *Ndst4* mutant rat (founder #21) with wild type rat.

The PAM was underlined in red. The substituted nucleotides were shown in blue.


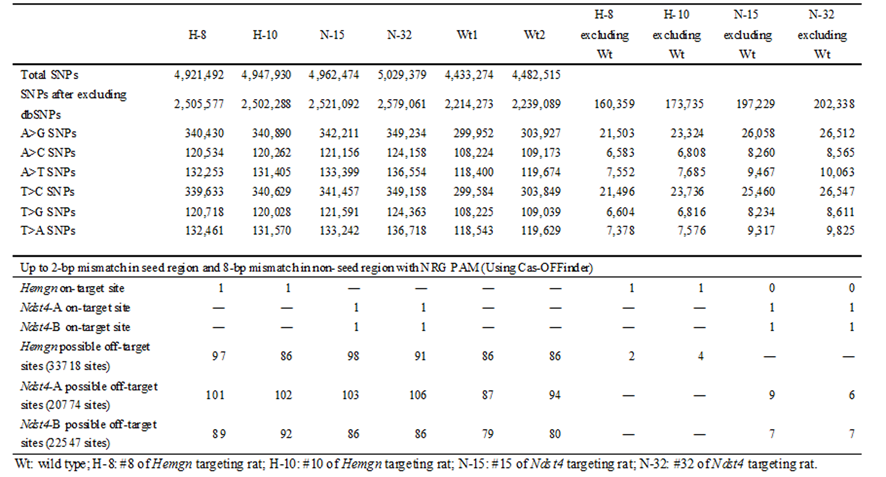


**Figure S6 Whole genome sequencing of *Hemgn* mutant, *Ndst4* mutant and wild-type rat.**

Summary of whole genome sequencing analysis. The mutant rat and wild type rat were separately sequenced using illumina Hiseq ×10. After filtering out the SNPs in database and excluding wild-type SNPs, a total of 160,359 SNPs obtained in H-8 (*Hemgn* targeting rat), 173,735 SNPs in H-10 (*Hemgn* targeting rat), 197,229 SNPs in N-15 (*Ndst4* targeting rat), 202,338 SNPs in N-32 (*Ndst4* targeting rat). Next we compared the DNA sequences at the remaining SNP sites with the on target sequences. Among 33,718 proto-spacer adjacent motif (PAM)-containing sites that differ from the *Hemgn* on-target site by up to 2 bp mismatch in the seed region and 8 bp mismatch in the non-seed region, just 2 sites in H-8, and 4 sites in H10 were identified as potential off-target sites. Among 20,774 PAM-containing sites that differ from the *Ndst4*-A on-target site, just 9 sites in N-15A, and 6 sites in N-32A were identified as potential off-target sites. Among 22547 PAM-containing sites that differ from the *Ndst4*-B on-target site, just 7 sites in N-15B, and 7 sites in N-32B were identified as potential off-target sites.

**Supplementary Tables**

**Supplementary Table 1 Oligonucleotides for sgRNA plasmid preparation**

| R-Hemgn-E- gRNA up | 5’- TAGGAGGTATTGGTACCTGCTG -3’ |
| --- | --- |
| R-Hemgn-E- gRNA dw | 5’- AAACCAGCAGGTACCAATACCT -3’ |
| R-Ndst4-E-A-gRNA up | 5’- TAGGATGTATCAATAGGCTTGA -3’ |
| R- Ndst4-E-A-gRNA dw | 5’- AAACTCAAGCCTATTGATACAT -3’ |
| R-Ndst4-E-B-gRNA up | 5’- TAGGCTGACACTATCCTTGGAC -3’ |
| R- Ndst4-E-B-gRNA dw | 5’- AAACGTCCAAGGATAGTGTCAG -3’ |

**Supplementary Table 2 Primers for amplifying and sequencing of on target fragments**

| Name | Sequence (5’-3’) | Amplicon |
| --- | --- | --- |
| R-Hemgn–F1 | CACACACACACACACATACACTTG | 536 bp |
| R-Hemgn–R1 | CCTTCGTTGGTGTGTTTGAG |  |
| R-Ndst4-F1 | ATGGTTACATGAATGTTTGGGAAG | 1251 bp |
| R-Ndst4–R1 | CGTGTATGCATTTGTATATCTGAGC |  |

**Supplementary Table 3 Summary of mutations in *Ndst4* sgRNA-B targeting sites**

| Triple mutation  (15^th^, 13^th^,10^th^ ) | Dual mutation  (15^th^,13^th^ ) | Dual mutation  (15^th^, 10^th^ ) | Single mutation  (15^th^) | Single mutation  (13^th^) |
| --- | --- | --- | --- | --- |
| #7, #18, #20, #22, #32 | #1, #2, #5, #6, #14, #15, #21, #24, #27 | #3 | #4, #8, #10, #11-13, #16, #17, #19, #23, #25, #28, #30 | #29 |

Data based on Fig. S3.

**Supplementary Table 4 Primers for amplifying and sequencing of on-target and off-target fragments**

| Name | Sequence (5’-3’) | Amplicon (bp) |
| --- | --- | --- |
| R-Hemgn –F2 | CCGAAAGAGACAAGGGAATG | 261 |
| R-Hemgn –R2 | GCTTTGTCCCTTCGTTGG |  |
| R-Ndst4-A-F2 | CCCTTGTCGAAACTACTGCA | 319 |
| R-Ndst4 -A-R2 | TCCCGGTTCCATGAGTCCAT |  |
| R-Ndst4-B-F2 | AACCATTCTACCTACCAGCCAGTG | 285 |
| R-Ndst4 -B-R2 | CATCCTTGTGCCCTCCTTCC |  |
| Hemgn-OFF1-F1 | GCTAGGGTGACACCAATGTG | 364 |
| Hemgn-OFF1-R1 | GAAGGCAGCTCTGTGGCTAT |  |
| Hemgn-OFF2-F1 | ACTCCAGGTCTACCTCCCAGTG | 353 |
| Hemgn-OFF2-R1 | GGTGTCCCAATCCCTCCACT |  |
| Hemgn-OFF3-F1 | TAGGGAGATAGATGATTGCTGC | 322 |
| Hemgn-OFF3-R1 | TAACCTTCAAATACCTCCTTGC |  |
| Hemgn-OFF4-F1 | GTGCAGAACGACCGACGAGA | 347 |
| Hemgn-OFF4-R1 | TCCTTTCTACCCACCAACTT |  |
| Hemgn-OFF5-F1 | GCCAGCAAACAACAACATAC | 386 |
| Hemgn-OFF5-R1 | AAGCTAACCCAGGCACGACT |  |
| Hemgn-OFF6-F1 | GGGACAGTATGCTAACCTTCAC | 355 |
| Hemgn-OFF6-R1 | ATCAGTTTGGACCCACTTCTT |  |
| Hemgn-OFF7-F1 | ATCCCTCATTTGCTCCCTAA | 320 |
| Hemgn-OFF7-R1 | CATGGCTACTTCAGTCTCCC |  |
| Hemgn-OFF8-F1 | AAAGACCAGAGCACGGAAGA | 370 |
| Hemgn-OFF8-R1 | TTAGAGCCTCGGACAAACCT |  |
| Hemgn-OFF9-F1 | TGATGAAGGCATGGAGGTTC | 325 |
| Hemgn-OFF9-R1 | CAGCCAGTAGCAGACTTGTTGA |  |
| Hemgn-OFF10-F1 | CTGCTGAGCCACTTCAAGAGCTTCC | 306 |
| Hemgn-OFF10-R1 | GCTGCCCACTAACTCATCGTCACCTA |  |
| Hemgn-OFF11-F1 | GCTGATGAACTGGCTGAACG | 371 |
| Hemgn-OFF11-R1 | TGCCTTTGTGGAGGTATTGG |  |
| NDST-A-OFF1-F1 | ACGTGGAGGCTGGAGGTC | 479 |
| NDST-A-OFF1-R1 | TTCCAGATCCTTTTCATGACACTAA |  |
| NDST-A-OFF2-F1 | CTAGGAAAGTGGGTGGAGTTAGAAAT | 477 |
| NDST-A-OFF2-R1 | TGACCCTGTAACTTGTGCCTTC |  |
| NDST-A-OFF3-F1 | AGCCTGTGACCCTGTAACTTGT | 474 |
| NDST-A-OFF3-R1 | TGTAGGAAAATGGGTGGAGTTAGA |  |
| NDST-A-OFF4-F1 | TCCTGATCCTCCTGCTTTTACC | 496 |
| NDST-A-OFF4-R1 | GCAGTACACCCTCCTCATAACATAG |  |
| NDST-A-OFF5-F1 | CCCTAACTGTCTTCATTTTTCTGG | 549 |
| NDST-A-OFF5-R1 | ACCAATTACAAGCTAACATTCCAAAG |  |
| NDST-A-OFF6-F1 | CTCACCTTTGTAGATGGAAGCC | 504 |
| NDST-A-OFF6-R1 | CAATGCTCACTTCCTCAATGTCT |  |
| NDST-A-OFF7-F1 | CCAGGGACACAGATTCAAGTTC | 496 |
| NDST-A-OFF7-R1 | TGACTGTCTCCTGTATCCCCAC |  |
| NDST-A-OFF8-F1 | CTTGCTCAGCCTGCTTTCTTAG | 487 |
| NDST-A-OFF8-R1 | AGAGTAGTTCTTAGAGGCAATTGC |  |
| NDST-A-OFF9-F1 | AGTAGCTGCTAAACTCTGACAAACG | 499 |
| NDST-A-OFF9-R1 | TTCATTGTGGAGAAGATACTGCG |  |
| NDST-A-OFF10-F1 | GAAGCCTCAGAGACCACAAAAG | 455 |
| NDST-A-OFF10-R1 | GCTTGCTTGATGGGAAACAG |  |
| NDST-A-OFF11-F1 | CACCTTCCTCAGTCCTGTTACC | 495 |
| NDST-A-OFF11-R1 | CCCTTCTCCTCTCACACTCG |  |
| NDST-A-OFF12-F1 | CCTCCTGCTTTTACCTCTCAAGTA | 489 |
| NDST-A-OFF12-R1 | GCAGTACACCCTCCTCATAACATAG |  |
| NDST-A-OFF13-F1 | ACTTGGTTCTGAATTTCTCCTCCT | 495 |
| NDST-A-OFF13-R1 | CTGATGGGTTGTTCTTATGGG |  |
| NDST-A-OFF14-F1 | CACAGATTCAAGTTCCCCAGA | 465 |
| NDST-A-OFF14-R1 | CCTCACTACCGGGCTTAACTTC |  |
| NDST-B-OFF1-F1 | ATAGGGCTAGAATTCCTGTTTTACC | 323 |
| NDST-B-OFF1-R1 | GTGCCTGCCTTCACCCTATA |  |
| NDST-B-OFF2-F1 | TCGAGGTCCTGCATTGTAAAG | 345 |
| NDST-B-OFF2-R1 | CTTCCTTCTGCTGTGATAAAATATGA |  |
| NDST-B-OFF3-F1 | AGAAAGGAGCTAGATTTTATTACCGA | 285 |
| NDST-B-OFF3-R1 | GGATGGGGATAGGATTCACTTC |  |
| NDST-B-OFF4-F1 | CCTAACTGTTACCTCTCAATGCTCA | 336 |
| NDST-B-OFF4-R1 | CGGCCAGTAACAGGTTTCG |  |
| NDST-B-OFF5-F1 | TTACCCTGATTGGCTCTATTCC | 275 |
| NDST-B-OFF5-R1 | AAGGATTAAAATGAGGCAGCC |  |
| NDST-B-OFF6-F1 | GATGCCACCAACTGAGAAACTC | 285 |
| NDST-B-OFF6-R1 | TCCCATCTCTAAACATGCATTTTAT |  |
| NDST-B-OFF7-F1 | CACAGTTGCTATCCTCATCCAC | 339 |
| NDST-B-OFF7-R1 | AGTGCCTCAGTCTGTCCAGG |  |
| NDST-B-OFF8-F1 | TTAGAATCACGTGGGAGACAGTC | 300 |
| NDST-B-OFF8-R1 | CAAAGGTTTATTTTGGCTCCC |  |
| NDST-B-OFF9-F1 | CTTGGAGCAGCTAGTCACAACC | 295 |
| NDST-B-OFF9-R1 | GTGTTACAAATTTATCTCCCAAAGTCA |  |
| NDST-B-OFF10-F1 | CATGCAAGTTAGTGTTAGAATAGGGC | 331 |
| NDST-B-OFF10-R1 | CACCCTATACCCTCACGTCATATAG |  |
| NDST-B-OFF11-F1 | AGACTCCCAGGACCCAACAA | 272 |
| NDST-B-OFF11-R1 | GCAGTAGTGTTGGGGTTTGC |  |
| NDST-B-OFF12-F1 | GGCTGATTCAGGACCATAGTTTC | 335 |
| NDST-B-OFF12-R1 | AGGTCAGAGGTAAAGGAACAGCT |  |
| NDST-B-OFF13-F1 | ATCTGTCATCAAACGCAATCACT | 287 |
| NDST-B-OFF13-R1 | AGATGATCTCATTTAACCGGAAGC |  |
| NDST-B-OFF14-F1 | GTGGTCCAGGATTTGTTGTTC | 365 |
| NDST-B-OFF14-R1 | CCTGTCTCTCATACTCACACCCA |  |

**Supplementary Table 5 Potential off-target sites of *Hemgn* and *Ndst4*-A/B sgRNAs in the rat genome**

| **No.** | **Gene** | **sequence** | **Chromosome** | **Position** | **Direction** |
| --- | --- | --- | --- | --- | --- |
| On target | Hemgn | GGAGGTATTGGTACCTGCTGAGG | 5 | 59242149-59242127 | - |
| Hemgn-OT1 | Intergenic region | tGAtGTATTGGTACaTGCTGGGG | 8 | 80688333-80688311 | + |
| Hemgn-OT2 | [Ptprc](https://www.ncbi.nlm.nih.gov/gene/19264) | GGAGGTAaTGGaACCTtCTGTGG | 3 | 149146740-149146718 | - |
| Hemgn-OT3 | Intergenic region | GGAGGTATTGGaAgCTGCTGGGG | 3 | 164860781-164860803 | - |
| Hemgn-OT4 | [Scyl2](https://www.ncbi.nlm.nih.gov/gene/213326) | GGgGtTATTGGTACCTGCTGTGG | 7 | 21246594-21246572 | - |
| Hemgn-OT5 | Intergenic region | GGAGGaATTaGTtCCTGCTGGGG | 1 | 168962442-168962464 | + |
| Hemgn-OT6 | [Tkfc](https://www.ncbi.nlm.nih.gov/gene/225913) | GGAGGTAcTGGggCCTGCTGAGG | 1 | 204735371-204735349 | - |
| Hemgn-OT7 | Intergenic region | GGAGcTATTGGTAaCTaCTGGGG | 1 | 250799221-250799199 | - |
| Hemgn-OT8 | Intergenic region | GGAGagATTGGTtCCTGCTGTGG | 17 | 6031798- 6031820 | + |
| Hemgn-OT9 | Intergenic region | GGAGGTtTTGGTACtTGaTGTGG | 14 | 8248890-8248912 | + |
| Hemgn-OT10 | [Sowahb](https://www.ncbi.nlm.nih.gov/gene/78088) | GGAGGTAcaGGgACCTGCTGGGG | 14 | 14432012-14432034 | + |
| Hemgn-OT11 | Intergenic region | GGAGGTATTGGTtCCTtaTGGGG | 9 | 34264260-34264238 | - |
| Ndst4-A-On | Ndst4 | GGATGTATCAATAGGCTTGATGG | 2 | 206169455-206169433 | - |
| Ndst4-A-OT1 | Intergenic region | GGAcGTATCAATcGGaTTGATGG | 3 | 5549017- 5549039 | + |
| Ndst4-A-OT2 | Intergenic region | GTgTGTATCAATAGGCTTGgAGG | 4 | 149966516- 149966494 | - |
| Ndst4-A-OT3 | Intergenic region | GTgTGTATCAATAGGCTTGgAGG | 4 | 150179776- 150179798 | + |
| Ndst4-A-OT4 | Intergenic region | GGgTGTATaAAcAGGCTTGAAGG | 5 | 114544834- 114544856 | + |
| Ndst4-A-OT5 | Intergenic region | GGATGTATCcATAaGCaTGAAGG | 1 | 207696762- 207696740 | - |
| Ndst4-A-OT6 | Intergenic region | GGATcTcTCAATAGGCTTGgGGG | 2 | 168717040- 168717018 | - |
| Ndst4-A-OT7 | Intergenic region | GGATGTATCAtTAaGCTTGAGGG | 11 | 66856014- 66855992 | - |
| Ndst4-A-OT8 | Intergenic region | tGATGTATaAATAGGACTTGATGG | 12 | 25409194- 25409217 | + |
| Ndst4-A-OT9 | Intergenic region | GGATG-ATCAGTAAGCTTGAGGG | 3 | 95135952- 95135931 | + |
| Ndst4-A-OT10 | Intergenic region | GGATGTATACAATgGGCTaGATGG | 3 | 39518455- 39518478 | + |
| Ndst4-A-OT11 | Creb5 | GGATGgATCAAT-GGaTTGATGG | 4 | 77584154- 77584133 | + |
| Ndst4-A-OT12 | Intergenic region | GG-TGTATaAAcAGGCTTGAAGG | 5 | 114544835- 114544856 | + |
| Ndst4-A-OT13 | Kcnip4 | GGA-GaATCAATAGtCTTGAGGG | 14 | 60765933- 60765912 | - |
| Ndst4-A-OT14 | Intergenic region | GGGATGTATCAtTAaGCTTGAGGG | 11 | 66856015- 66855992 | - |
| Ndst4-B-On | Ndst4 | GGCTGACACTATCCTTGGACAGG | 2 | 206170117- 206170139 | + |
| Ndst4-B-OT1 | Intergenic region | tGCTGACACTATCCTTtGAaGGG | 7 | 59527087- 59527109 | + |
| Ndst4-B-OT2 | Asap1 | GGgTGACACTATtCTTGGgCAGG | 7 | 92523055- 92523077 | - |
| Ndst4-B-OT3 | Intergenic region | GGCTcACACTATCCTgGGACTGG | 4 | 121829145- 121829167 | + |
| Ndst4-B-OT4 | Sox5 | GGCTGgCACTATCaTTGGACCGG | 4 | 165558465- 165558443 | - |
| Ndst4-B-OT5 | Rhobtb3 | GGCTGACACTgTCCTTGaACTGG | 2 | 1840065- 1840087 | + |
| Ndst4-B-OT6 | Ndst3 | GaCTGACACTgTCCTTGGACAGG | 2 | 204265039- 204265017 | + |
| Ndst4-B-OT7 | Tbkbp1 | GcCTGACACTcTCCTTGGAgTGG | 10 | 80885090- 80885112 | + |
| Ndst4-B-OT8 | Intergenic region | GGCTGgCACCATCCaTGGACTGG | 20 | 23139921- 23139943 | + |
| Ndst4-B-OT9 | Intergenic region | GGaTGACACTATTCCTTaGACAGG | 3 | 40969463- 40969440 | - |
| Ndst4-B-OT10 | Intergenic region | G-CTGACACTATCCTTtGAaGGG | 7 | 59527088- 59527109 | + |
| Ndst4-B-OT11 | Intergenic region | GG-TGGCcCTATCCTTGGACTGG | 4 | 170116354- 170116333 | - |
| Ndst4-B-OT12 | Park2 | GaCTGACACTATCCTTGGATaTGG | 1 | 44986990- 44986967 | - |
| Ndst4-B-OT13 | Intergenic region | GGCTG-CACTgTCCTTGGAaAGG | 19 | 48501919- 48501940 | + |
| Ndst4-B-OT14 | Intergenic region | GGCTGACACTgTCCTTG-AaGGG | 17 | 73399224- 73399245 | + |

**Supplementary Table 6 Potential off-target sites of *Hemgn* and *Ndst4*-A/B identified by WGS**

|  | Location | | | | Seqence | Mismatch |
| --- | --- | --- | --- | --- | --- | --- |
| H-10  (*Hemgn*) | chr1 | 270416384 | 270416406 | + | acAttaAgTaGgACCTGCTGTAGG | 8 |
|  | chr6 | 11946942 | 11946964 | - | GcAGaccaTGGTtCCaGCTGGAG | 7 |
|  | chr8 | 8183091 | 8183113 | - | cagGtccaaGGTgCCTGCTGAGG | 9 |
|  | chr10 | 146599 | 146621 | - | catctTtaTGGTcCCTGCaGTAG | 9 |
| H-8  (*Hemgn*) | chr1 | 270416384 | 270416406 | + | acAttaAgTaGgACCTGCTGTAGG | 8 |
|  | chr3 | 104638993 | 104639015 | - | aGgGtatTTGaTgCCTGCTGTGG | 7 |
| N-15  (*Ndst4*-A) | chr1 | 74965822 | 74965843 | + | tGAaaaATgAATAGGCaTGAAGG | 6 |
|  | chr1 | 193827334 | 193827356 | - | taccactgtAATAGGCTTtAAGG | 10 |
|  | chr4 | 70759111 | 70759133 | - | tctcccATCAcTAGGCTTtAAAG | 8 |
|  | chr8 | 21915325 | 21915347 | - | aacccccgCcATtGGCTTGAAAG | 10 |
|  | chr9 | 14550125 | 14550146 | + | cacTGataCAATgGGCTaGAGAG | 8 |
|  | chr11 | 74485785 | 74485806 | + | aGgcagcTCAAcAGGCTgGAGGG | 8 |
|  | chr16 | 68757418 | 68757440 | - | cagaGaccCAATAGGaTTaATGG | 9 |
|  | chr18 | 37480954 | 37480975 | + | aGtTaTcTCAATAtGgTTGATAG | 6 |
|  | chr20 | 17500818 | 17500840 | + | ctTtggaCAATAGGCTaGAAGGG | 7 |
| N-32  (*Ndst4*-A) | chr1 | 74965822 | 74965843 | + | tGAaaaATgAATAGGCaTGAAGG | 6 |
|  | chr4 | 21113717 | 21113738 | + | aGtaaagaCAATAaGCTTcAGAG | 9 |
|  | chr4 | 70759111 | 70759133 | - | tctcccATCAcTAGGCTTtAAAG | 8 |
|  | chr15 | 25069492 | 25069514 | - | GtgTGggTCAtTAGGCTgGAGGG | 6 |
|  | chr16 | 68757418 | 68757440 | - | cagaGaccCAATAGGaTTaATGG | 9 |
|  | chr20 | 17500818 | 17500840 | + | ctTtggaCAATAGGCTaGAAGGG | 7 |
| N-15  (*Ndst4*-B) | chr1 | 235997331 | 235997353 | + | GaTttgAaTATCCTTGGcCCAGG | 7 |
|  | chr1 | 236593497 | 236593519 | + | GtCcttaAgTATCCcTGGACAGG | 7 |
|  | chr3 | 123987856 | 123987877 | + | aGgTGgCgCTATCCcTGGgCTGG | 6 |
|  | chr10 | 19981725 | 19981747 | - | ctCTGAgtCTATaCTTGGAaGAG | 6 |
|  | chr10 | 76653820 | 76653842 | - | tcCacACACTcTtCTTGGACCAG | 6 |
|  | chr15 | 35243332 | 35243353 | + | ccagGtggaTATCCTTGGtCTGG | 9 |
|  | chr20 | 7495909 | 7495931 | - | acaTacagCTtTCtTTGGACTGG | 9 |
| N-32  (*Ndst4*-B) | chr1 | 235997331 | 235997353 | + | GaTttgAaTATCCTTGGcCCAGG | 7 |
|  | chr1 | 236593497 | 236593518 | + | GtCcttaAgTATCCcTGGACAGG | 7 |
|  | chr2 | 19975296 | 19975318 | - | aGgaacatCTATCCgTGcACCAG | 9 |
|  | chr10 | 19981725 | 19981747 | - | ctCTGAgtCTATaCTTGGAaGAG | 6 |
|  | chr11 | 64942565 | 64942587 | - | tttctAtACTgTCCTTGGAaCAG | 8 |
|  | chr14 | 12339223 | 12339244 | + | aaCgaggcCTgTCaTTGGACGAG | 9 |
|  | chr16 | 25989980 | 25990002 | - | aatgaggcCTgTCaTTGGACGAG | 10 |

**Supplementary References**

1. Gaudelli*,* N. M. *et al.* Programmable base editing of A*T to G*C in genomic DNA without DNA cleavage*.* *Nature* **551**, 464-471 (2017).

2. Ma, Y. *et al.* Generating rats with conditional alleles using CRISPR/Cas9*.* *Cell Res* **24**, 122-125 (2014).

3. Ma*,* Y. *et al.* Building Cre Knockin Rat Lines Using CRISPR/Cas9*.* *Methods Mol Biol* **1642**, 37-52 (2017).
